# An Overview of the Effectiveness of Corticoids in Croup: A Systematic Literature Review

**DOI:** 10.7759/cureus.46317

**Published:** 2023-10-01

**Authors:** Neyla Garzon Mora, Arturo P Jaramillo, Ruth Briones Andriuoli, Sol Torres, Jhoanny C Revilla, Denisse Moncada

**Affiliations:** 1 Medicine, Universidad Católica de Santiago de Guayaquil, Guayaquil, ECU; 2 General Practice, Universidad Estatal de Guayaquil, Machala, ECU; 3 Pediatrics, Universidad Católica de Santiago de Guayaquil, Guayaquil, ECU; 4 Medical School, Universidad Católica de Santiago de Guayaquil, Guayaquil, ECU; 5 Medicine, Universidad del Zulia, Maracaibo, VEN

**Keywords:** laryngotracheobronchitis, seasonal change, adrenaline, corticoid, croup

## Abstract

Croup, also known as laryngotracheobronchitis, frequently leads to blockages in the upper respiratory tract in young children, presenting symptoms, such as a raspy voice, a distinctive cough, and noisy breathing during inhalation. Despite being a condition that often resolves on its own, it puts considerable strain on healthcare resources due to regular doctor visits, emergency room usage, and occasional hospital stays. Research focused on larger populations suggests that only a small percentage of children with croup end up requiring hospital admission for their condition. In line with the Preferred Reporting Items for Systematic Reviews and Meta-Analyses (PRISMA) 2020 guidelines, we executed a meticulous systematic review by scouring databases, such as PubMed, Google Scholar, and the Cochrane Library. A total of 10 articles met our inclusion criteria and were selected for in-depth analysis. These scholarly works provided substantive insights into the pharmacological agents deployed in the treatment of croup. From a clinical standpoint, the management of croup is highly contingent on the patient's hemodynamic status. Our review discerned a pronounced preference for corticosteroids as the primary therapeutic intervention over other alternatives, which are largely relegated to second-line or emergency applications. Interestingly, we found negligible differences among the various corticosteroid treatment options in terms of statistical significance, underscoring their broad utility in ameliorating the condition. In addition to corticosteroids, our review also explored other therapeutic options, such as heliox, nebulized adrenaline, and even natural interventions, such as exposure to outdoor cold air. The efficacy of these treatments demonstrated variable results, reinforcing the notion that while they may be useful in specific circumstances, they are not universally applicable or as robustly effective as corticosteroids. Given the preponderance of evidence favoring corticosteroids, further research is warranted to solidify their status as the first-line treatment in different medical settings, be it inpatient hospitals, outpatient clinics, or even for home-based care. Such studies will not only add a layer of confidence in current medical practice but could also potentially optimize treatment protocols, contributing to improved patient outcomes. Therefore, advancing our understanding of the effectiveness of corticosteroids as the cornerstone of croup management remains an area of paramount scientific and clinical importance.

## Introduction and background

Croup is a common respiratory ailment affecting young children, and it is a significant reason for pediatric healthcare visits, making up nearly 15% of all trips to healthcare facilities for respiratory issues in this age group. The symptoms are quite distinctive and include a hoarse or raspy voice, a cough that has a barking quality, difficulty with inhalation, and varying degrees of discomfort in breathing that can escalate quickly [1].

A thorough review carried out by the Cochrane Collaboration compiled information from 38 different studies focused on how effective glucocorticoids are in treating croup symptoms. The findings were quite revealing; the use of glucocorticoids was shown to have a favorable impact on the Westley croup score, a measure of croup severity, particularly within six to 12 hours of administration [2]. Children treated with glucocorticoids generally had shorter stays in emergency departments, exhibited milder symptoms of viral croup, and required less frequent use of epinephrine. Furthermore, these children had fewer instances of needing to return for additional treatment or being readmitted to healthcare facilities [3].

A number of studies have also been designed to explore the most effective ways to administer glucocorticoids, examining various delivery methods, such as injections (parenteral), orally ingested medications, and nebulized (inhaled) forms. Across various levels of croup severity, all these methods, whether it was intramuscular, intravenous, oral, or inhaled, proved to be effective. Support for these findings also comes from a randomized controlled trial (RCT) performed in Iran [4,5]. In another study focusing on outpatient care, oral dexamethasone was compared with oral prednisolone in the treatment of 87 children with mild to moderate croup symptoms. The study revealed that there were no significant differences in the need for additional healthcare for croup in the 11 days following the initial treatment, nor in any other measured outcomes [6].

Hospitalized children with croup often receive multiple rounds of steroids, a practice that likely stems from an absence of inpatient-specific guidelines and a perception that multiple dosages may sustain the alleviation of symptoms and avert their return after initial improvements [7-9]. A recent investigation involving 327 hospitalized pediatric patients with croup discovered that about 48% were treated with multi-day steroid regimens. As a result, oral corticosteroids are usually the preferred choice for treatment, given their ease of administration and less invasive nature compared to intramuscular injections [7]. Moreover, the oral route is often favored over nebulizer treatments because it is generally more effective, easier to administer, and more cost-efficient [7].

Corticosteroids have gained wide acceptance as a standard treatment in emergency departments, including our own, and are prescribed routinely for all diagnosed cases of croup [7]. Their established safety and efficacy have made them crucial in decreasing the rate of hospitalizations, the duration of hospital stays, the number of follow-up medical visits, ICU admissions, and even the necessity for endotracheal intubation in severe cases [7].

In our systematic review, we aim to provide a well-rounded assessment of the utility of corticoids for treating croup in various healthcare settings, ranging from inpatient hospital wards and emergency rooms to at-home care and outpatient clinics. We synthesized data from each of these settings to offer a comprehensive perspective on this important treatment strategy.

## Review

Methodology

In conducting our systematic review, we adhered to rigorous methodological standards outlined in the Preferred Reporting Items for Systematic Reviews and Meta-Analyses (PRISMA) guidelines, thereby ensuring the comprehensiveness and transparency of our approach and findings. Our screening process entailed querying three reputable databases: PubMed, the Cochrane Library, and Google Scholar. We employed advanced search techniques, such as Medical Subject Headings (MeSH) keyword searching and Boolean logic, to ensure a robust capture of relevant literature. In addition, only free full-length papers were included to ensure comprehensive data extraction and interpretation.

Quality appraisal of the selected articles was performed using the Assessment of Multiple Systematic Reviews (AMSTAR) checklist, a tool that is recognized for its robustness in evaluating the methodological quality of systematic reviews. Moreover, to scrutinize the level of bias in clinical trials included in our review, we applied the Cochrane risk-of-bias assessment tool, thereby fortifying the credibility of our systematic literature review (SLR).

Study Duration and Search Strategy

On June 4, 2023, we used the databases PubMed, Cochrane Library, and Google Scholar to extract articles relevant to this review. To conduct our search on PubMed, we used the regular search tool. We looked for the following MeSH keywords: ("Croup/diet therapy"[Majr:NoExp] OR "Croup/drug therapy"[Majr:NoExp] OR "Croup/prevention and control"[Majr:NoExp] OR "Croup/rehabilitation"[Majr:NoExp] OR "Croup/therapy"[Majr:NoExp]) AND "Parainfluenza Virus 2, Human/drug effects"[Majr:NoExp] AND ("Adrenal Cortex Hormones/administration and dosage"[Majr:NoExp] OR "Adrenal Cortex Hormones/blood"[Majr:NoExp] OR "Adrenal Cortex Hormones/metabolism"[Majr:NoExp] OR "Adrenal Cortex Hormones/pharmacokinetics"[Majr:NoExp] OR "Adrenal Cortex Hormones/pharmacology"[Majr:NoExp] OR "Adrenal Cortex Hormones/therapeutic use"[Majr:NoExp]) AND ("Croup/drug therapy"[Majr:NoExp] OR "Croup/prevention and control"[Majr:NoExp] OR "Croup/therapy"[Majr:NoExp]).

Inclusion and Exclusion Criteria

We included observational studies, RCTs, systematic reviews, traditional reviews, meta-analysis journals, and other articles in English. We included studies carried out after 2013 focusing on the different outcomes of corticoids in patients with croup and excluded editorials, perspectives, case reports, peer reviews, gray literature, unpublished studies, and animal studies (Table 1).

**Table 1:** Inclusion and exclusion criteria RCTs: randomized controlled trials

	Inclusion criteria	Exclusion criteria
Language	Literature published in the English language	Literature published in languages other than English
Type of study	Observational studies, RCTs, systematic reviews, traditional reviews, and meta-analysis journals	Editorials, perspectives, case reports, peer reviews, gray literature, unpublished studies, and animal studies
Year of Ppublishing	Articles published after 2013	Articles published before 2013
Content of the study	Articles with content relevant to the research question	Articles focusing on treatments that are not corticosteroids
Population	Children and adults	Pregnant patients

Results

Search Results and Selection of Articles

**Figure 1:**
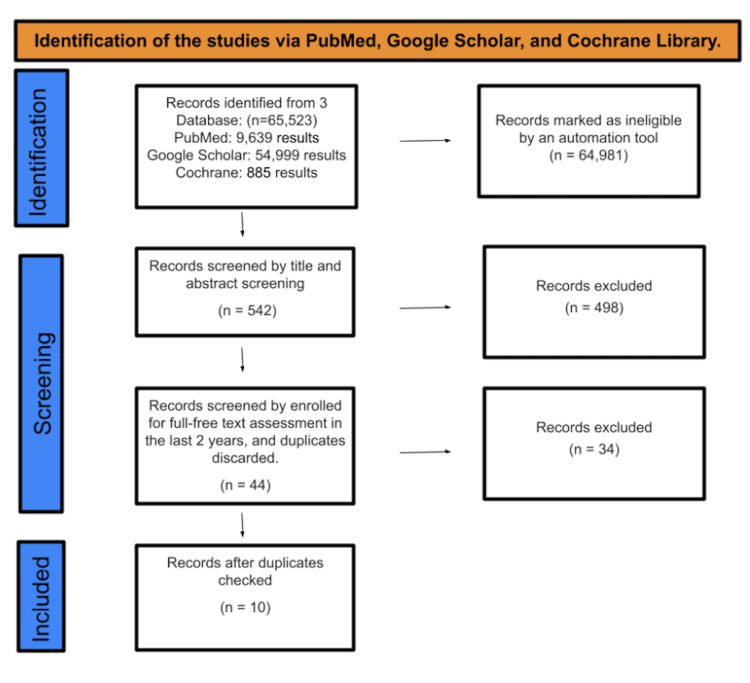
PRISMA flow diagram

After searching in PubMed, Google Scholar, and the Cochrane Library, a total of 65,523 studies were identified. An automatic program flagged 64,981 people as ineligible. A total of 542 research were screened for title and abstract, with 498 articles being discarded. The remaining 44 publications were picked by a full-free text review throughout the past 10 years, and after removing duplicates, which resulted in the omission of 34 studies, just 10 were recruited for the final data collection. The full PRISMA flow diagram of the article selection method is shown in Figure 1.

Table 2 shows an in-depth description of the articles we decided to use.

**Table 2:** Findings from the data extraction RCT: randomized clinical trial; SLR: systematic literature review; WCS: Westley croup score; LOS: length of stay; KASCH: King Abdullah Specialized Children's Hospital; AE: adverse effects

Author	Year of publication	Study design	Quality tool	Primary research	Outcome evaluation
Siebert et al. [10]	2023	RCT	Cochrane risk-of-bias assessment tool	Participated in a study involving children between the ages of three months and 10 years with a WCS higher than 2, who visited a specialized pediatric emergency room	A total of 118 children were randomly divided into two groups: one exposed to outside cold air and the other to indoor room air.
Asif et al. [11]	2023	RCT	Cochrane risk-of-bias assessment tool	Included 226 kids in this study who had a WCS of 2 or more. The study showed that 0.15 mg/kg of oral dexamethasone effectively lowered the overall croup score, although it did not statistically affect respiratory and pulse rates or oxygen levels.	Conducted a study with children aged six months to six years, admitted to five different American pediatric hospitals from July 2014 to June 2016.
Tyler et al. [12]	2023	RCT	Cochrane risk-of-bias assessment tool	Conducted a study with children aged six months to six years, admitted to five different American pediatric hospitals from July 2014 to June 2016	Considerable variations in dexamethasone dosing and LOS across different hospitals were noted.
Alqahtani et al. [13]	2022	RCT	Cochrane risk-of-bias assessment tool	Reviewed electronic health records from KASCH in Riyadh, Saudi Arabia, for all croup patients between 2014 and 2018	The timing of dexamethasone administration did not significantly affect recovery or relapse rates, but chronic illnesses did significantly affect relapse rates.
Moraa et al. [14]	2021	RCT	Cochrane risk-of-bias assessment tool	Included three RCTs with a total of 91 children aged between six months and four years	Heliox appears to be no more effective than 30% oxygen for mild croup, but as effective as 100% oxygen administered with one or two doses of adrenaline.
Fernandes et al. [15]	2019	RCT	Cochrane risk-of-bias assessment tool	Children under six years with acute respiratory issues were given either inhaled or systemic corticosteroids for up to 14 days.	Short-term high-dose corticosteroid use does not seem to increase AEs in different organ systems.
Gates et al. [16]	2018	RCT	Cochrane risk-of-bias assessment tool	Conducted a study on children aged 0 to 18 years with croup, comparing the effects of glucocorticoids alone or combined with other treatments to placebos or alternative medicines	Glucocorticoids reduced croup symptoms within two hours, decreased hospital stays, and reduced return visits for care, altering the conclusions of a prior review.
Elliott et al. [17]	2017	SLR	Cochrane risk-of-bias assessment tool	Multiple RCTs and SLRs have largely focused on the effectiveness of dexamethasone as an oral remedy for croup in kids.	In situations where dexamethasone is not accessible, prednisolone seems to be a suitable alternative for treating mild to moderate croup.
Johnson et al. [18]	2014	SLR	Cochrane risk-of-bias assessment tool	Evaluated 19 studies and graded the evidence to assess the effectiveness and safety of various interventions, such as corticosteroids, nebulized budesonide, oral prednisolone, heliox, humidification, and nebulized adrenaline	We present information relating to the effectiveness and safety of the following interventions: corticosteroids, nebulized budesonide, oral prednisolone, heliox, humidification, and nebulized adrenaline.
Garbutt et al. [19]	2013	RCT	Cochrane risk-of-bias assessment tool	The study aimed to evaluate the efficacy of prednisolone at a dose of 2 mg/kg/day for three days, compared to a single dose of dexamethasone at 0.6 mg/kg and two placebo doses.	No significant differences in treatment outcomes were observed for either the child or the parent between the two croup therapies.

After assessing 10 RCTs for quality, we attributed seven "+" to seven of them and six "+" to one. We considered these studies high quality and decided to include them in our systematic review. The results are presented in Table 3. The AMSTAR criteria is shown in Table 4.

**Table 3:** Quality assessment of RCTs RCTs: randomized controlled trials Siebert et al. [10]; Asif et al. [11]; Tyler et al. [12]; Alqahtani et al. [13];  Moraa et al. [14]; Fernandes et al. [15]; Gates et al. [16]; Garbutt et al. [19]

Studies	Random sequence generation (selection bias)	Allocation concealment (selection bias)	Blinding of participants	Blinding of personnel/care providers (performance bias)	Blinding of outcome assessor (detection bias)	Incomplete outcome data (attrition bias)	Selective reporting (reporting bias)	Other biases	Overall
Siebert et al. [10]	+	+	+	+	+	+	+	-	7/8
Asif et al. [11]	+	+	+	+	+	+	+	-	7/8
Tyler et al. [12]	+	+	+	+	+	+	+	-	7/8
Alqahtani et al. [13]	+	+	+	+	+	+	+	-	7/8
Moraa et al. [14]	+	+	+	+	+	+	+	-	7/8
Fernandes et al. [15]	+	+	+	+	?	+	+	-	6/8
Gates et al. [16]	+	+	+	+	+	+	+	-	7/8
Garbutt et al. [19]	+	+	+	+	+	+	+	-	7/8

**Table 4:** AMSTAR criteria AMSTAR: A Measurement Tool to Assess Systematic Reviews

AMSTAR Criteria	Elliott et al., 2017 [17]	Johnson et al., 2014 [18]
Priori design provided	Yes	Yes
Duplicate study selection, data extraction present	Yes	Yes
Comprehensive literature search performed	Yes	Yes
Was the status of publication used as inclusion criteria	Yes	Yes
A list of inclusion and exclusion studies provided	Yes	No
Characteristics of inclusion studies provided	Yes	Yes
Quality of inclusion studies included and documented	No	No
Quality of inclusion studies used appropriately in forming conclusions	No	No
Appropriate methods used to combine studies	Yes	Yes
Likelihood of publication bias assessed	No	Yes
Conflict of interest included	Yes	Yes
Final score assigned	8/11	8/11

Discussion

In this systematic review, we aim to provide an overview of corticosteroids used in patients with croup in various settings, including hospitals, emergency rooms, and outpatient clinics. We will also offer brief insights into the differences between the drugs used and which one is more popular and effective in treating the condition. It is an intriguing topic, especially when considering the diverse medical settings in which croup is managed. The efficacy of corticosteroids can vary widely depending on numerous factors, making such a review quite valuable for medical professionals

In a comprehensive analysis of multiple research studies focused on the management and treatment of croup in pediatric patients, several key insights emerge.

Siebert et al. conducted a single-center, open-label RCT to investigate the therapeutic effects of exposure to cold outdoor air for 30 minutes in children with croup symptoms. Their findings offer initial clinical evidence suggesting that brief exposure to cold air, with temperatures below 10°C, may alleviate the severity of croup symptoms, particularly those of moderate intensity. This study further showed that the efficacy of this treatment was comparable to the administration of oral dexamethasone, a corticosteroid, particularly when considering symptom improvement or resolution at 60 minutes post-treatment [10].

Another RCT by Asif et al. highlighted that corticosteroids, such as dexamethasone, could enhance symptom relief within six hours of administration, with lasting effects up to 12 hours. The study also demonstrated reduced healthcare utilization measures, including the necessity for nebulized adrenaline, the duration of emergency department stays, and the frequency of readmissions and return visits [11].

Tyler et al. performed a multisite prospective cohort study on children aged between six months and six years across five US children's hospitals. They identified substantial variations in the administration of multiple dexamethasone doses across the participating hospitals and found that about one-third of the admitted children had already received steroids before hospital arrival, possibly due to persistent symptoms [12].

In a study by Alqahtani et al., the temporal parameters for administering dexamethasone were scrutinized. Their data did not indicate any statistically significant differences between early and late administration of the drug in terms of either recovery or relapse rates. However, they did note a marginally better recovery rate and fewer relapses in cases where the medication was administered later [13].

Moraa et al. conducted an RCT to assess the efficacy of heliox, a mixture of helium and oxygen, for treating children with mild to moderate croup. Their investigation did not conclusively demonstrate the superiority of heliox over other oxygen administration methods [14]. A systematic review by Fernandes et al., involving 85 studies and over 11,000 pediatric patients, revealed that short-term corticosteroid usage did not significantly increase adverse events across multiple organ systems [15].

Moreover, Gates et al. provided evidence that glucocorticoids, specifically budesonide and dexamethasone, were effective in alleviating croup symptoms within two hours, with effects lasting for at least 24 hours [16]. Elliott et al. suggested that while prednisolone may be effective initially, it appears less successful than dexamethasone in preventing symptom recurrence [17]. However, Johnson et al. proposed that small, short doses of prednisolone could be a viable alternative for the treatment of mild to moderate croup when dexamethasone is not available [18].

Lastly, a study by Garbutt et al. evaluated the comparative efficacy of intramuscular dexamethasone versus nebulized budesonide. They found no qualitative differences between the two treatment modalities in terms of hospital admission rates, the need for additional treatments, or changes in the Westley croup score. Their study also emphasized that a higher dose of corticosteroids yielded more significant symptom relief compared to a placebo [19]. Garbutt et al. further discovered that a three-day course of oral prednisolone was equivalent to a single oral dose of dexamethasone for treating mild or moderate croup, in terms of healthcare utilization and symptom duration [19,20].

In sum, these collective findings suggest a multifaceted approach to the management and treatment of croup in pediatric populations, emphasizing the comparable efficacy of various corticosteroids and other alternative treatments. Further studies are warranted to refine treatment guidelines.

## Conclusions

Our analysis reveals that corticosteroid treatments significantly improved patient outcomes as measured by the Westley croup score, without a concomitant increase in adverse effects. In addition, our findings indicate a reduction in the utilization of second-line therapies, such as nebulized adrenaline, both in hospital settings and during outpatient walk-in visits. This carries the dual benefit of shortening hospital stays and reducing disease recurrence rates. It is also worth noting that while the dosages of corticosteroids varied across studies, the therapeutic efficacy was largely consistent. Similarly, when comparing different corticosteroids, such as budesonide and fluticasone, against alternative treatments, such as nebulized adrenaline and heliox, the improvement in patient symptoms maintained statistical significance.
